# Alteration in gut microbiota caused by time‐restricted feeding alleviate hepatic ischaemia reperfusion injury in mice

**DOI:** 10.1111/jcmm.14069

**Published:** 2018-12-26

**Authors:** Jinjun Ren, Dandan Hu, Yilei Mao, Huayu Yang, Wenjun Liao, Wei Xu, Penglei Ge, Hongbing Zhang, Xinting Sang, Xin Lu, Shouxian Zhong

**Affiliations:** ^1^ Peking Union Medical College Hospital Beijing China; ^2^ Sun Yat‐sen University Cancer Center Guangzhou China; ^3^ Second Affiliated Hospital of Nanchang University Nanchang China; ^4^ The First Affiliated Hospital of Zhengzhou University Zhengzhou China; ^5^ Institute of Basic Medical Sciences and School of Basic Medicine, Chinese Academy of Medical Sciences Beijing China

**Keywords:** gut microbiome, hepatic ischaemia reperfusion injury, time‐restricted feeding

## Abstract

Time‐restricted feeding (TRF), that is, no caloric intake for 14‐16 hours each day leads to favourable nutritional outcomes. This study is the first to investigate TRF through a surgical perspective verifying its efficacy against liver ischaemia reperfusion (I/R) injury. We randomly assigned 100 10‐week‐old wild‐type male C57BL/6 mice into two feeding regimens: TRF and ad libitum access to food. Main outcomes were evaluated at 6, 12 and 24 hours post‐I/R surgery after 12 weeks of intervention. TRF group demonstrated minor liver injury via histological study; lower serum levels of liver enzymes, glucose and lipids; higher concentrations of free fatty acid and β‐hydroxybutyrate; decreased oxidative stress and inflammatory biomarkers; as well as less severe cell apoptosis and proliferation. Further exploration indicated better gut microenvironment and intestinal epithelial tight junction function. TRF employed its positive influence on a wide spectrum of biochemical pathways and ultimately revealed protective effect against hepatic I/R injury possibly through adjusting the gut microbiota. The results referred to a strong indication of adopting better feeding pattern for surgical patients.

## 1 INTRODUCTION

Time restricted feeding (TRF) allows food consumption within 8‐10 hours period per day, and no caloric intake is permitted during the rest 14‐16 hours. No restriction is imposed on the quantity and nutritional constitution of food. Accumulated evidence has proved that TRF is a promising intervention against the worldwide trend of obesity.^1^ TRF is also associated with reductions in serum total cholesterol, triglycerides (TG), glucose, insulin, interleukin 6 (IL‐6), tumour necrosis factor‐α (TNF‐α), as well as with improvements in insulin sensitivity.^2^ Feeding behaviour is predominantly dictated by our inherent circadian clock, and this timing system and other factors like food availability, social habits and convenience jointly decide the way a life entity eats. The comprehensive benefits of TRF are considered to root in its compatibility with our inborn biological clock.^3^

Liver plays a critical role in the basal metabolism and progression of many diseases regarding its close relationship with food intake.^4^ And the gut continuously encounters the environmental influence brought by the diet content and schedule. Therefore, the gut microbiota as a whole is responsible for maintaining a micro‐homeostasis.^5^ Previous studies have investigated the influence of feeding pattern on liver diseases and the hepatic injury resistance capacity. Ischaemia reperfusion (I/R) injury model is among the most commonly used prototypes, as it mimics one of the major complications introduced by liver transplantation, liver resection, trauma, etc. Multiple strategies have been investigated as potential protective approaches against I/R injury, including helium preconditioning^6^ medications,^7^ supplement of saturated fatty acids^8^ and extracorporeal liver assist device to exchange albumin and remove endotoxin.^9^ The influence of fasting on metabolic status has been proved to be modest in magnitude but broad in extent. Taken together, various indicators of systemic biochemistry dictated the boost in hepatic anti‐I/R capacity caused by fasting.^10^ Some studies underlined the anti‐inflammatory properties of fasting, and pre‐operative fasting may be an effective medical order to protect the liver against I/R injury.^11^, ^12^ Meanwhile, mechanistic studies disclosed the association between acute liver impairment with increased oxidative stress and inflammatory reaction. And fasting protected the liver from these two factors through its regulatory actions on ketone body levels.^13^ However, surgical patients could hardly tolerant a malnutrition status, which largely brought down the feasibility of fasting. TRF, on the other hand, is often regarded as an alternative, or a better approach to fasting, making it promising to investigate the efficacy of this feeding pattern against liver I/R injury.

## 2 EXPERIMENTAL PROCEDURES

### 2.1 Animals and diets

We performed all animal experiments under the Institutional Animal Care and Use Committee (IACUC) guidelines of the Salk Institute. C57BL/6 male mice at 10 weeks of age were entrained to a 12:12 light‐dark cycle. Normal food chow (GB14924.3‐2010 Standard: 29% protein, 13% fat, 58% carbohydrate) [by reference: LabDiet‐5010] was used, and drinking water was sterile filtered. We randomly assigned 100 mice into two regimens: TRF: all food consumption was restricted within an 8‐10 hours period (23 pm next day 7 am) per day, no caloric intake was allowed for the rest 14‐16 hours. Ad libitum (AL): ad libitum access to food. Neither feeding pattern imposed limitation on the total amount of food the mice subjects consumed. Dietary intervention lasted for 12 weeks before hepatic I/R operation was performed.

### 2.2 Hepatic ischaemia reperfusion injury model

Partial (70%) warm ischaemia was performed as described.^14^ In brief, after dissecting the portal triad, the blood supply to the left and median liver lobes was occluded with a microvascular clamp for 60 minutes, before removing the clamp to initiate reperfusion. Sham group underwent identical surgical procedures except vascular occlusion. Blood, liver and colon faecal were harvested after 6, 12 and 24 hours from post‐operative mice. As the operation time is closely associated with the severity of liver injury, we finished the first surgical procedure for mice between 8 to 9 am. Both groups went through an hour of fasting before the operation began.

### 2.3 Serum biochemistry

Automatic biochemistry analyzer was used to detect the concentration of liver transaminases, blood glucose, TG, cholesterol and free fatty acids. Enzyme‐linked immunosorbent assay kits were used to test the levels of TNF‐α, IL‐1β, IL‐6, IL‐10 and β‐hydroxybutyrate.

### 2.4 Hepatic parameters measurement

#### 2.4.1 Triglycerides

Liver powder was homogenized in isopropanol, and TG concentration was measured using an enzymatic assay, and data were normalized to liver weight. Glycogen: liver samples were weighed and digested, precipitated and hydrolysed, and results were normalized to wet weight of liver.^15^ β‐hydroxybutyrate concentration in the liver was assayed spectrophotometrically. Myeloperoxidase activity was detected by the spectrophotometric method. Total Superoxide Dismutase (SOD) Assay Kit with WST‐8, Cellular Glutathione Peroxidase (GPx) Assay Kit, Lipid Peroxidation malondialdehyde (MDA) Assay Kit, Quantitative Assay Kit and Glutathione (GSH) Colorimetric Detection Kit were used to detect the concentration of SOD, GPx, MDA, H_2_O_2_ and GSH respectively.

#### 2.4.2 Western blotting

Western blot method was applied to detect the levels of liver NF‐KB, MAPKs signal pathway protein, NLRP3 inflammatory protein complexes and LPS/TLR4 signal pathway related proteins. Liver specimens were homogenized in lysis buffer (RIPA). Proteins were separated by sodium dodecyl sulfate‐polyacrylamide gel electrophoresis and transferred to nitrocellulose membranes. Membranes were incubated with primary antibodies at 1:1000 dilution with 5% bull serum albumin. After overnight incubation, horseradish peroxidase‐conjugated second antibodies were added for 1 hour at room temperature. Glyceraldehyde 3‐phosphate dehydrogenase was used for normalization.

#### 2.4.3 Histological and TUNEL analysis

Liver specimen harvest was performed at post‐surgical time 6, 12 and 24 hours, and samples were fixed and embedded in paraffin. Sections were stained with haematoxylin‐eosin (H&E). Light microscopic examination of liver specimens was performed with a light microscope. Suzuki's criteria of hepatic ischaemia/reperfusion injury were used to assess the severity of liver injury in both groups.^16^

**Table:** 

Score	Congestion (%)	Vacuolization (%)	Necrosis (%)
0	None	None	None
1	Minimal (10)	Minimal (10)	Single cell necrosis
2	Mild (11‐30)	Mild (11‐30)	Mild (<30)
3	Moderate (31‐60)	Moderate (31‐60)	Moderate (<60)
4	Severe (>60)	Severe (>60)	Severe (>60)

Terminal deoxynucleotidyl transferase‐mediated dUTP nick‐end labelling (TUNEL) was subsequently performed on paraffin sections using a commercially available in situ cell death detection kit (Roche Diagnostics, Basel, Switzerland). The TUNEL index was determined by counting the positive and negative stained hepatocytes in each of the 10 fields of vision. Within each vision, 100 hepatocytes were counted. And the TUNEL index = positively TUNEL‐stained cells/1000, n = 6).

#### 2.4.4 Metagenomics DNA extraction and 16S rRNA sequencing

Six mice from each group were killed before and at 6, 12 and 24 hours after I/R surgery. To ensure the similarities, within group was not caused by co‐housing of mouse subjects, every mouse sample was randomly chosen from a different cage. Faeces samples were isolated from colon and flash frozen. DNA from the samples was obtained after multiple procedures including suspension, digestion, lysis, precipitation, wash and resuspension. Based on the hypervariable V1‐V3 region, 16S rRNA gene sequence tags were generated using the 454 pyrosequencing platform. Alpha diversity, beta diversity and operational taxonomic unit‐based classification were analysed.

### 2.5 Statistical analyses

Data were analysed by Stata/MP 14.0. All normally distributed data were displayed as the mean ± SD. Pairwise comparisons between two groups were performed by the Student's *t* test. A value of *P* < 0.05 indicated statistical significance.

## 3 RESULTS

### 3.1 Time‐restricted feeding retarded weight gain

Bodyweight was monitored weekly for 12 weeks, and TRF was proved to down‐regulate the weight gain. TRF mice had significant lower bodyweight, compared to the mice that had ad libitum access (AL) to food: 31.13 ± 0.76 versus 33.14 ± 0.81 g, *P* < 0.05, n = 50.

### 3.2 TRF mitigated liver ischaemia reperfusion injury

**Figure 1:**
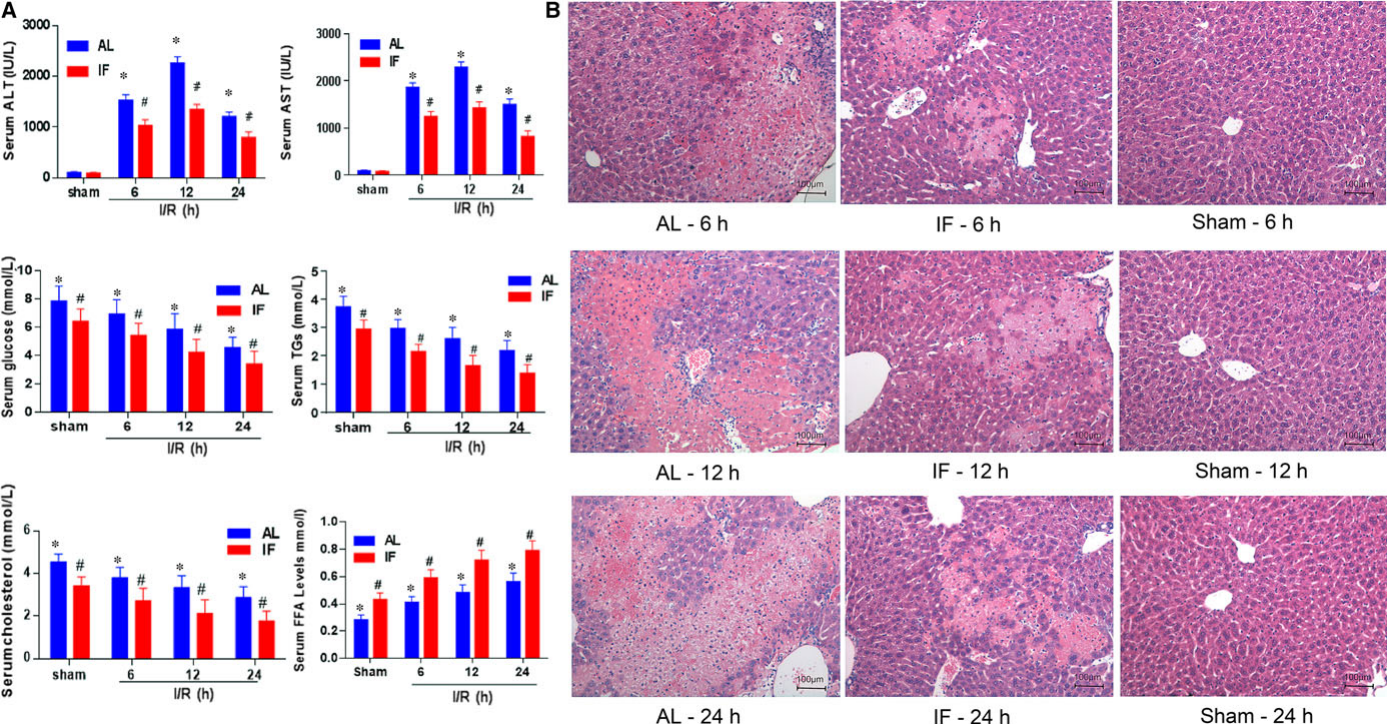
Indicators of ischaemia reperfusion injury. (A) Biochemistry profiles reflecting the severity of liver impairment, including serum ALT, AST, glucose, TGs, cholesterol and FFA levels were measured at the first 6, 12 and 24 h after hepatic portal occlusion surgery respectively. Sham operations were performed in the control group. *P < 0.05 indicated significant difference between ischaemia reperfusion group and sham group. # *P* < 0.05 was tagged if statistical significance was detected between AL and TRF groups at a given time. If not specified, n = 6. (B). Representative H&E‐stained histological sections of liver at 6, 12 and 24 h after portal vein blockage and sham operation (10 × 20 magnification). AL: ad libitum; TRF: Time‐restricted feeding; ALT: Alanine transaminase; AST: aspartate transaminase; TGs: triglycerides; FFA: free fatty acid

According to the results of biochemistry profile, hepatic portal occlusion exerted a major damage to the liver, with all indicators significantly different with the sham group at all time points. Regarding the variations between AL and TRF, a strong protective effect was revealed by TRF, namely the serum concentrations of ALT, AST, glucose, TGs, cholesterol were decreased in the TRF group, while FFA level was increased, which all together provided evidence for the better liver function in this group (Figure 1A). And the positive effects emerged shortly after the I/R intervention, and remained at least 24 hours after surgery. Similar conclusion was drawn from pathological studies: According to the Suzuki's criteria of hepatic ischaemia/reperfusion injury, average score of IF and AL group 6, 12, 24 hours after surgery were 5.00 ± 1.17 versus 7.00 ± 1.80 (*P* = 0.00), 4.67 ± 1.21 versus 7.57 ± 1.41 (*P* = 0.00), 5.00 ± 0.98 versus 7.30 ± 1.12 (*P* = 0.00) respectively (Figure 1B).

### 3.3 TRF altered energy expenditure pattern

**Figure 2:**
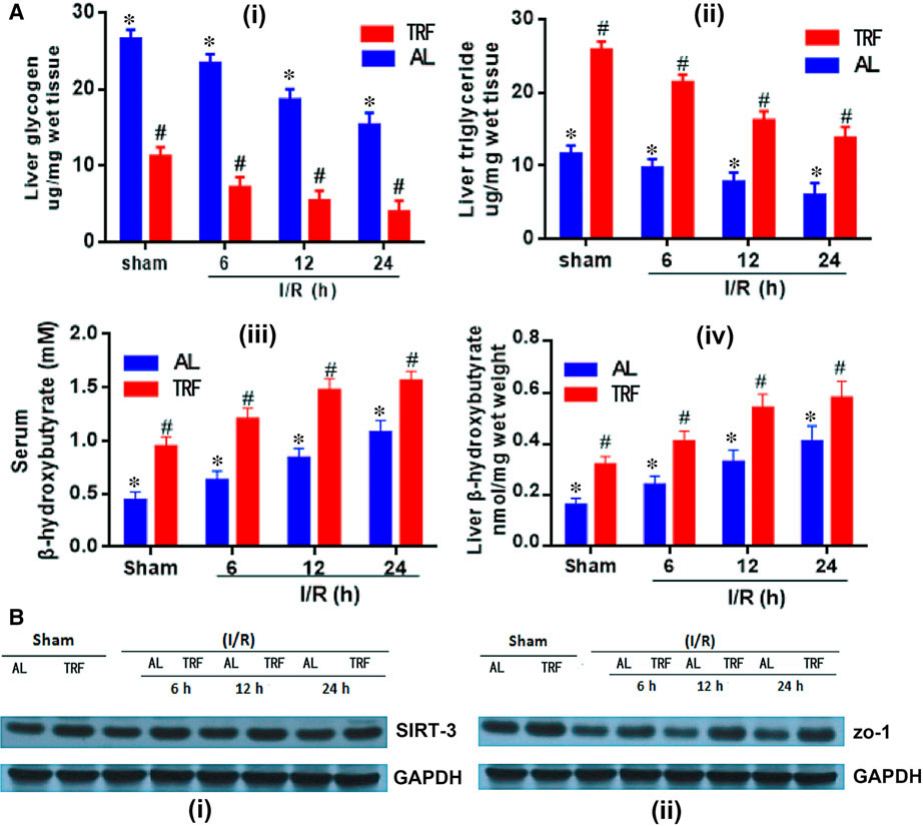
Alterations of liver energy source. (A) Concentrations of liver glycogen, liver triglyceride, serum β‐hydroxybutyrate and liver β‐hydroxybutyrate were shown by different timing and feeding model respectively, *P < 0.05 indicated significant difference between ischaemia reperfusion group and sham group. #*P* < 0.05 was tagged if statistical significance was detected between AL and TRF groups, n = 6. (B) Western blot was applied to show the increase of SIRT‐3 and zo‐1 proteins caused by TRF dietary pattern. GAPDH was used as the internal reference. AL: ad libitum; TRF: Time‐restricted feeding; zo‐1: zonula occludens‐1; GAPDH: glyceraldehyde 3‐phosphate dehydrogenase

Energy expenditure differed greatly under AL and TRF feeding mode: TRF facilitated the conversion from glucose metabolism to ketogenic metabolism. The lower level of liver glycogen, and increased concentration of liver TG, serum β‐hydroxybutyrate and liver β‐hydroxybutyrate together supported this conclusion (Figure 2A). SIRT‐3 protein, encoded by *SIRT‐3* gene, is implicated in regulation of metabolic process, in this regard, it has a role in the adaptive thermogenesis of adipose tissue. Higher concentration of SIRT‐3 was detected at all times in TRF mice. Similar was true regarding zo‐1 protein (Figure 2B). The up‐regulation of zo‐1 was a positive indicator of protection against gut flora shift through tight junction of intestinal epithelial cells.

### 3.4 Effects on the apoptosis and regeneration of hepatocytes

**Figure 3:**
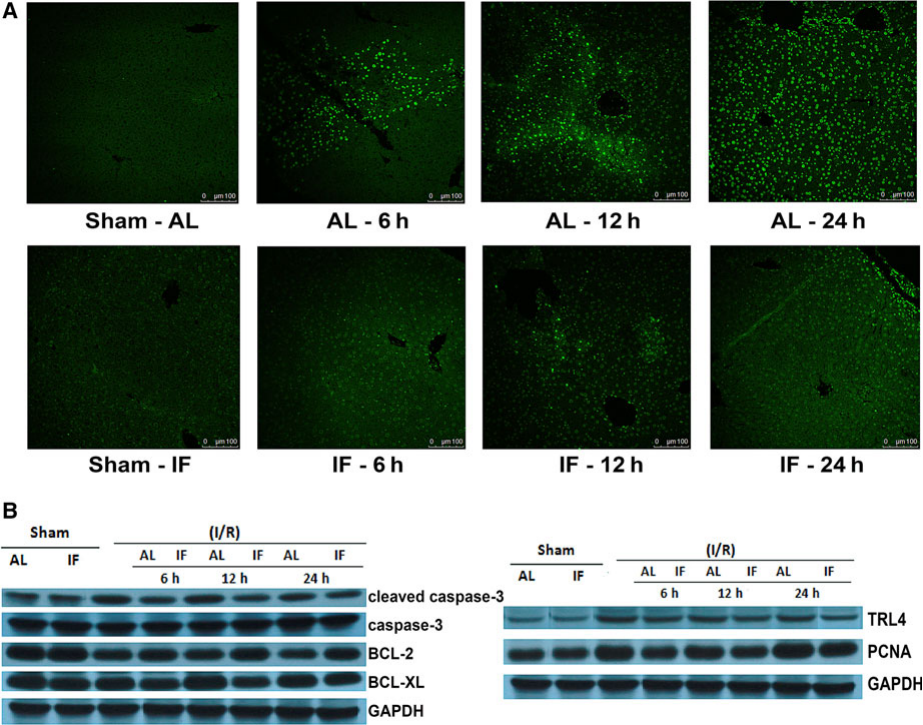
Hepatocytes apoptosis and regeneration. (A) Represented TUNEL‐stained histological liver sections of sham operation, as well as 6, 12 and 24 h after ischaemia reperfusion surgery by AL and TRF groups, n = 4. (B) Western blot results of some proteins participating in the process of cell apoptosis and regeneration. GAPDH was used as the internal reference, n = 5. AL: ad libitum; TRF: Time‐restricted feeding; TUNEL: terminal‐deoxynucleotidyl transferase mediated nick‐end labelling; Bcl‐2: B‐cell lymphoma 2; Bcl‐Xl: B‐cell lymphoma‐extra large; GAPDH: glyceraldehyde 3‐phosphate dehydrogenase; TRL4: toll‐like receptor 4; PCNA: Proliferating cell nuclear antigen

Cell apoptosis is programmed cell death, which can be revealed by TUNEL‐stain in situ. Figure 3A demonstrated that hepatocytes apoptosis was predominantly inhibited by TRF. The TUNEL index of IF and AL group 6, 12, 24 hours after surgery were 25.2 ± 9.4 versus 47.1 ± 10.8 (*P* = 0.035), 27.2 ± 5.6 versus 58.7 ± 8.5 (*P* = 0.0003), 25.3 ± 8.9 versus 71.0 ± 8.1 (*P* = 0.000) respectively. Western blot technology further proved that the concentration of cleaved caspase‐3, an apoptosis‐promoting protein, was down‐regulated in the TRF group. However, the levels of caspase‐3 were proximal between groups. Bcl‐2 and Bcl‐XL are both anti‐apoptosis proteins, and they had higher expression in the TRF group. TLR4 is a transmembrane protein, whose activation leads to pro‐inflammatory cytokine signalling. TRF mice showed lower levels of TLR4, which was in accord with the assumption that TRF protected the liver from I/R injury. PCNA acts as a scaffold to recruit proteins involved in DNA replication, DNA repair, chromatin remodelling and epigenetics. Decreased level of PCNA protein in TRF group indicated lower level of cell regeneration and repair in this group (Figure 3B). Both cell apoptosis and regeneration were relatively more unrestricted in the AL group.

### 3.5 TRF inhibited oxidative stress and inflammatory reaction

**Figure 4:**
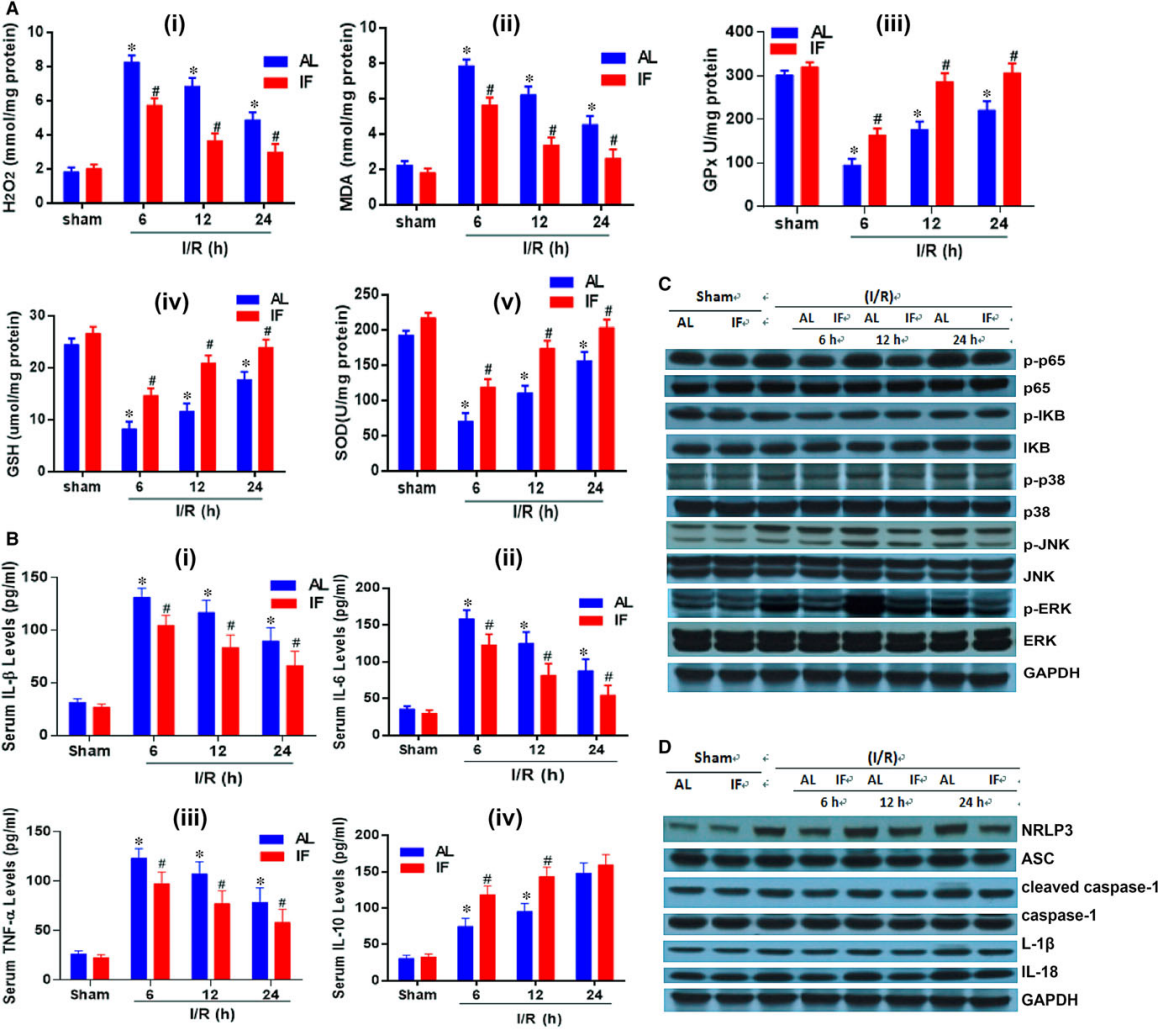
Biomarkers of oxidative stress and inflammatory reactions. (A) The levels of oxidative stress biomarkers in the liver measured at 6, 12, 24 h post‐operation, and comparison with the sham group. Biomarkers included: H_2_O_2_, MDA, GPx, GSH, and SOD. (B) Serum concentration of inflammatory factors including TNF‐α, IL‐1β, IL‐6, and IL‐10. *P < 0.05 indicated significant difference between ischaemia reperfusion group and sham group. #*P* < 0.05 was tagged if statistical significance was detected between AL and TRF groups, n = 6. (C) Western blot showing titre of proteins involved in ischaemia reperfusion injury pathways. (D) Demonstration of inflammatory complex and proteins via Western blot technology. AL: ad libitum; TRF: time‐restricted feeding; MDA: malondialdehyde; GPx: glutathione peroxidase; GSH: glutathione; SOD: superoxide dismutase; TNF‐α: tumour necrosis factor‐ α, IL: interleukin; p‐p65: phospho‐P65‐nuclear factor (NF)‐κB; p‐IKB: phospho‐IκB; p‐p38: phospho‐p38; GAPDH: glyceraldehyde 3‐phosphate dehydrogenase; NLRP3: NACHT, LRR and PYD domains‐containing protein 3; ASC: apoptosis‐associated speck‐like protein containing a caspase recruitment domain

Time‐restricted feeding protects liver from severe I/R injury through inhibiting oxidative stress reactions and inflammatory reactions. Increased concentrations of H_2_O_2_ and MDA detected in the liver demonstrated that hepatic portal occlusion surgery led to aggregation of reactive oxygen species (ROS) and subsequently aggravate the liver impairment. TRF reduced the amount of ROS and down‐regulated the lipid peroxidation of hepatocyte membrane. GPx present in the mitochondrial matrix can scavenge H_2_O_2,_ 17 GSH is an antioxidant in almost all species, and SOD plays a critical role against oxidative inactivation of nitric oxide, thereby prevents peroxynitrite formation, and endothelial and mitochondrial dysfunction. These three biomarkers were decreased in TRF group, indicating protective effect was implemented upon liver through this feeding pattern (Figure 4A). Meanwhile, as one of the essential contributors to liver impairment, inflammation was suppressed by TRF. Pro‐inflammatory factors: TNF‐α, IL‐1β and IL‐6 showed lower levels in the TRF group, when compared with AL group at 6, 12 and 24 hours after surgery, while IL‐10, an anti‐inflammatory cytokine, had higher serum concentration in the TRF group than the IF group (Figure 4B). Identical result was drawn by Western blot, revealing decreased levels of p‐p65, p‐IκB, p‐p38, p‐JNK and p‐ERK, which were induced by I/R injury. No significant differences were detected between TRF and AL groups regarding total p65, IκB, p38, JNK and ERK. NLRP3 complex, ASC, cleaved caspase‐1, caspase‐1, IL‐1β and IL‐18 function as mediators in apoptosis and inflammation (Figure 4C). Concentrations of these proteins or complexes were cut down by TRF.

### 3.6 Protective mechanism might attribute to gut microbiota

**Figure 5:**
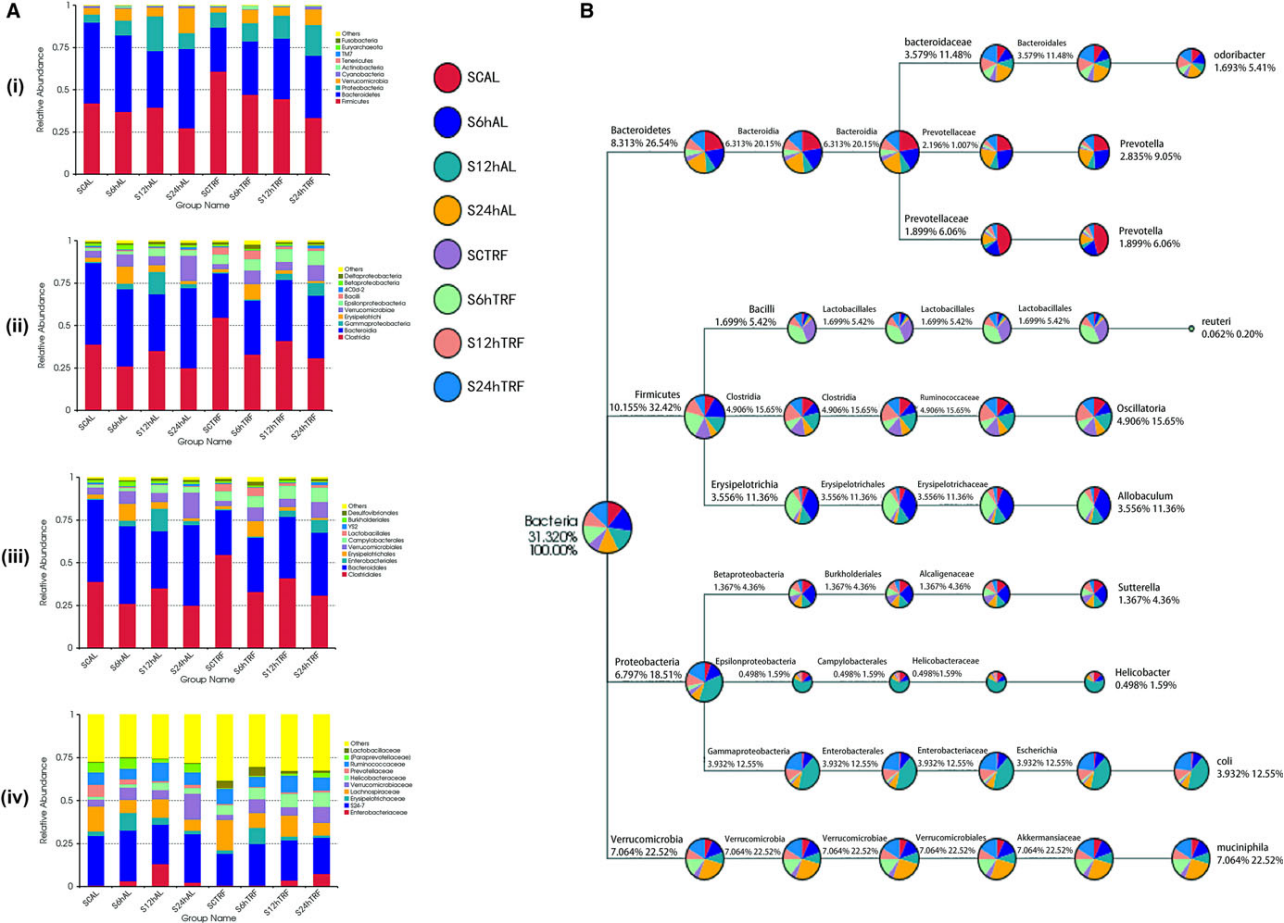
Gut microbiota. (A) Showed top 10 bacteria under phylum, class, order and family levels. Faeces samples were harvested at 6, 12 and 24 h after hepatic portal occlusion surgery, and controls were collected from sham operation group, n = 6. (B) Taxonomy tree revealing the relative abundance of the most frequent species detected. Different faeces samples collected at different timing from either AL or TRF group were indicated by different colours, and the sizes of circles represented the proportion of the corresponding micro‐organism, n = 6. SCAL: surgical control ad libitum; S6hAL: surgical 6 h ad libitum; S12hAL: surgical 12 h ad libitum; S24hAL: surgical 24 h ad libitum; SCTRF: surgical control time‐restricted feeding; S6hTRF: surgical 6 h time‐restricted feeding; S12hTRF: surgical 12 h time‐restricted feeding; S24hTRF: surgical 24 h time‐restricted feeding

Both the feeding pattern and timing after sample collection laid impacts on the composition of the intestinal ecosystem. Generally speaking, I/R was identified as a disastrous event to the gut microorganisms, while TRF reduces the damage (Figure 5A). Higher percentages of Firmicutes phylum, Clostridia and Bacilli class, Clostridiales and Lactobacillales order, Lachnospiraceae and Ruminococcaceae family, etc. were revealed in TRF mice samples, which could be hallmarks of a healthy gut (Figure 5B).

## 4 DISCUSSION

As a feeding schedule that imposes no restrictions on total caloric consumption or dietary structure, TRF has been profoundly proved to cause pleiotropic positive health outcomes. Besides the nutritional benefits, we now disclose its role in improving hepatic anti‐injury capacity: through alleviating oxidative stress, down regulating inflammatory reaction and inhibiting apoptosis and regeneration, TRF protected mice from hepatic I/R injury. Hepatic portal occlusion and reperfusion caused severe oxidative stress and inflammation, which were largely suppressed by TRF. Alarmed by endogenous danger signals released by stressed or dying hepatocytes, sentinel leucocyte populations constituted the proximal trigger for downstream pathways and reactions, which ultimately confered tissue destruction.^18^ Driven by NF‐Kb, NLRP3 cleaves and activates caspase‐1 and IL‐1β that in turn leads to a complex network of cellular reactions that initiates local and systemic inflammatory reactions.^19^, ^20^ These indicators were all decreased in the TRF group at 6, 12 and 24 hours after surgery. On the other hand, butyrate alleviates I/R‐induced hepatocytes injury by preventing NF‐kB activation,^21^ which was proved to have increased level in TRF mice analogous to the situation that fasting triggered. More specifically, the ketone metabolite β‐hydroxybutyrate blocked NLRP3 inflammasome‐mediated reactions.^22^ Meanwhile, toll‐like receptor 4 (TLR‐4) activation is implicated to play a key role in both inflammatory and fibrogenic pathways, thus contributing to liver disorders and complications of liver cirrhosis.^23^ However, though gut‐liver axis, TRF demonstrated its effect of down‐regulation of TLR‐4 level. Previous publications reported TRF groups had lower levels of IL‐6 and TNF‐α versus their respective control groups,^24^ which were more evident after I/R injury. And TRF mice showed higher serum IL‐10 concentration, which was considered to alleviate liver impairment via immune regulatory mechanism.^25^ Sirt3 increases nicotinamide adenine dinucleotide phosphate level and protects the liver from oxidative stress‐induced cell death, as well as enhances the mitochondrial glutathione antioxidant defence system,^26^, ^27^ thus our findings identify the increase of Sirt3 in TRF group supported the hypothesis that this feeding pattern was an essential player in the battle against oxidative stress. Also, antioxidant enzymes including SOD2 and Gpx1^12^ had higher concentrations in TRF mice. Inhibition of liver cells apoptosis and regeneration constituted another mechanism of protective effects of TRF. Terminal‐deoxynucleotidyl transferase mediated nick‐end labelling (TUNEL) stain revealed lower apoptotic rate in TRF group, which provided further evidence for relieved liver injury comparing to AL mice. Besides, as indicators of cell apoptosis, decreased levels of phosphor‐p38 and cleaved caspase‐3 were recognized in TRF mice.^28^

It is our assumption that through adaptation of the gut microbial ecosystem, TRF introduced positive changes in energy metabolism, inflammatory status and oxidative reactions, therefore contributed to protect the liver from I/R injury. This hypothesis was supported by the results of metabolic indicators, gut microbiota and intestinal tight junction regulators, etc. We confirmed that microbiome strongly impacts on the metabolome.^29^ Fasting led to insufficient gluconeogenesis, which was recognized by hypoglycaemia and increased fatty acid released from adipose tissue,^30^ in which case ketone bodies were produced in the liver and utilized in other tissues and organs.^31^ Our data showed that these were true regarding TRF, and ERK, which was believed to increase liver glycogen and decrease energy expenditure in obesity, showed lower concentration in TRF mice.^32^ Because the majority of the venous blood from the intestinal tract is drained into the portal circulation, the liver is hence the first organ to encounter absorbed nutrients and gut‐derived bacteria and pathogens. If the gut barrier is disrupted and allows microbial products and even viable bacteria to translocate from the digestive lumen to the live, potential damage is therefore introduced to the liver.^33^ In general, our data demonstrated TRF group was more inclined to ketogenic metabolic mode, one of the most evident proof was that the abundance of Firmicutes phylum in TRF mice largely exceeded that in AL mice. And probiotics were more frequent among gut flora from TRF mice as well. This result was highly in accordance with the fact that the injure brought by I/R operation was greatly shaded by TRF. It was believed that intestinal mucosa barrier function mediated the effect of gut microbiome on the body.^34^ And the intestinal epithelial tight junctions are charged with complex task of supporting transportation of nutrients while providing a barrier to microbial translocation, making the intestinal tract a unique organ where the permeability regulation is worth exploring,^35^ and even became a therapeutic target for a variety of diseases.^36^ Cytoplasmic adaptor proteins such as zo‐1 and some other protein components jointly seal the space between cell‐cell adhesion.^37^ The level of zo‐1 in TRF group was higher than that of the AL group, reflecting a more integrated barrier function under this feeding regimen.^38^ Previous studies have profoundly proved that gut microbiota was closely related to a wide spectrum of diseases including inflammatory bowel diseases, allergies, metabolic disorders and liver disease.^39^ Yet the correlation of gut flora and IR was not investigated before, which arose an insight into this feeding regimen and surgical intervention.

## 5 CONCLUSION

Time‐restricted feeding improved intestinal microenvironment and strengthened the intestinal epithelial tight junction, which provided favourable conditions leading to better energy metabolic status, inhibited inflammatory reaction, alleviated oxidative stress and suppressed hepatocytes apoptosis and proliferation. All these factors jointly contributed to an enhanced tolerance against I/R injury, indicating that adopting TRF regimen is highly recommended in patients scheduled for liver surgeries.

## ACKNOWLEDGEMENTS

This study was supported by CAMS Innovation Fund for Medical Sciences (2016‐I2M‐1‐001) and National High‐tech R&D Program (863 Program) (2015AA020303). We thank Qian Ma, Haihong Zhang from the Institute of Basic Medical Sciences, Chinese Academy of Medical Sciences for guidance of experimental techniques.

## CONFLICT OF INTEREST

All authors have no conflicts of interest to declare.

## AUTHORS’ CONTRIBUTION

YM, SZ: study supervision; JR (co‐first author): study design and procedures conduction; DH (co‐first author): data collection and manuscript drafting; HY, WX, PG: procedures conduction; WL: data collection and analysis; HZ: experiment methods supervision; XS, XL: critical revision for intellectual content.
